# Does the brain really know what word is coming next?

**DOI:** 10.7554/eLife.111163

**Published:** 2026-04-27

**Authors:** Richard J Antonello

**Affiliations:** 1 Zuckerman Institute, Columbia University New York United States

**Keywords:** language models, encoding models, predictive processing, None

## Abstract

Apparent neural encoding of future words may arise from the statistical structure of language itself, rather than from predictive computations in the brain.

**Related research article** Schönmann I, Szewczyk J, de Lange FP, Heilbron M. 2026. Stimulus dependencies – rather than next-word prediction – can explain pre-onset brain encoding in naturalistic listening designs. *eLife* **14**:RP106543. doi: 10.7554/eLife.106543.

Perhaps the most important open question in neuroscience is also the simplest: How does the brain learn? Over many decades of research, scientists have proposed several theories. Among the most prominent of these is the theory of “predictive coding”. In this theory, the brain learns by making predictions about the future, and then refining those predictions based on what has taken place (Rao and Ballard, 1999; de-Wit et al., 2010). If a prediction is incorrect, the brain can improve its understanding of the world by modifying its future predictions to better match what has actually happened. Conversely, if a prediction is accurate, this serves as a validation that the underlying model of the world that produced those predictions, the brain, has successfully learned about the world’s structure.

This theory is well regarded because it seems to align our experience of learning from our mistakes, and because artificial intelligence systems, such as neural networks, learn in a similar way (Goldstein et al., 2022; Caucheteux et al., 2023; Schrimpf et al., 2021). Moreover, there is also experimental evidence that supports predictive coding. This evidence includes the discovery by two independent groups of a “predictive representation” of words before they have been heard (Goldstein et al., 2022; Azizpour et al., 2025).

In these studies the researchers instructed individuals to listen to a story while recording their brain activity. It was found that word embeddings – mathematical representations of the information contained in a word – could be used to predict brain activity well before the individual heard the word. The researchers reasoned that if information about what will occur in the future was encoded in brain activity during the present, then this pre-onset encoding was strong, direct evidence of underlying predictive processes in the brain.

Now, in eLife, Inés Schönmann (Donders Institute for Brain, Cognition and Behaviour) and colleagues – Jakub Szewczyk, Floris de Lange and Micha Heilbron – report that they have reanalyzed this data, and found that it can be explained without assuming the theory of predictive coding to be true (Schönmann et al., 2026). They point out that the structure of language is itself loaded with a rich set of bidirectional dependencies that can inherently explain the phenomenon of pre-onset encoding on its own (Figure 1). For example, adjacent words can carry semantic meanings that are mutually interdependent (such as “Statue of Liberty”), mean more than the sum of their parts (such as “sour grapes”), or can express syntactic cues like tense or the presence of passive versus active voice.

**Figure 1.**
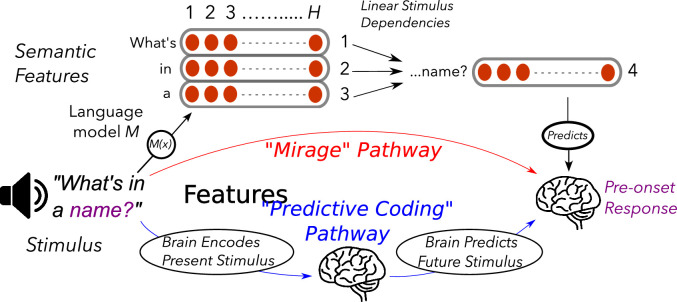
Two pathways for pre-onset encoding in the human brain. Pre-onset encoding is the phenomenon of apparent neural activations that occur before a stimulus – such as a word – is heard. For example, when hearing the words “what’s in a”, our brain will encode information related to the word “name” before we hear it. Researchers have proposed two pathways to explain this phenomenon. In the “mirage” pathway (top), the semantic features of each word are represented by a vector with H components. The structure of language means that there are bidirectional dependencies between the semantic features of the current stimulus (“what’s in a”) and the future stimulus (“name”). This means that the semantic features of the current stimulus can be used to predict semantic features of the future stimulus, which gives the appearance of pre-onset encoding of words in the brain. In the “predictive coding” pathway (bottom), the brain takes as input the current stimulus and, through internal computations, predicts the future stimulus, to later be checked against what actually occurs. This internal prediction of the future stimulus is encoded in the brain, which is then observed as pre-onset encoding of upcoming words.

Schönmann et al. used a passive control system to show that even in a hypothetical world where the brain encodes nothing but a fixed representation of its current stimulus, the dependencies intrinsic to language can work to present the appearance of next-word prediction. Whereas Goldstein et al. used a contextual embedding space derived from a language model – which is capable of implicitly encoding future words – to analyze their data, Schönmann et al. reanalyzed two different datasets using two systems that are known not to encode future words. These systems were: (i) a randomized lexical word embedding space which only captures the semantic properties of the current word; (ii) an auditory representation of the current stimulus that does not explicitly encode words at all.

In a reductio ad absurdum argument, they reason that if these explicitly non-predictive systems can show hallmarks of prediction similar to those used to provide evidence for a predictive representation in the brain, then the original methodology cannot be used to compellingly support the existence of explicitly predictive computations in the brain. They successfully demonstrate that these two non-predictive systems are indeed predictive representations in the same sense that the brain has been shown to be in prior studies. Furthermore, they show that methods that have been used in the past to try to correct for these stimulus dependencies, such as next-word residualization, are incapable of resolving the issue.

The work of Schönmann et al. – who are based at the Donders Institute, Jagiellonian University and the University of Amsterdam – joins a growing list of other studies (Antonello and Huth, 2022; Azizpour et al., 2025; Guest and Martin, 2023) that have sounded a note of caution about the use of encoding arguments for justifying mechanistic claims about the underlying nature of the brain. Although computational encoding methods are powerful new tools for understanding the brain, they are still correlational in nature and cannot be used in a vacuum to make definitive causative claims. Future work in this field should recognize these limitations and strive to supplement these analyses with causative experiments that directly address this important challenge.
